# The impact of the COVID-19 pandemic on daily rhythms

**DOI:** 10.1093/jamia/ocad140

**Published:** 2023-08-07

**Authors:** Nguyen Luong, Ian Barnett, Talayeh Aledavood

**Affiliations:** Department of Computer Science, Aalto University, Espoo, Finland; Department of Biostatistics, Epidemiology, and Informatics, University of Pennsylvania Perelman School of Medicine, Philadelphia, Pennsylvania, USA; Department of Computer Science, Aalto University, Espoo, Finland

**Keywords:** COVID-19, daily rhythms, mobile health, mixed effects models

## Abstract

**Objective:**

The COVID-19 pandemic has significantly impacted daily activity rhythms and life routines with people adjusting to new work schedules, exercise routines, and other everyday life activities. This study examines temporal changes in daily activity rhythms and routines during the COVID-19 pandemic, emphasizing disproportionate changes among working adult subgroups.

**Materials and Methods:**

In June 2021, we conducted a year-long study to collect high-resolution fitness tracker data and questionnaire responses from 128 working adults. Questionnaire data were analyzed to explore changes in exercise and work routines during the pandemic. We build temporal distributions of daily step counts to quantify their daily movement rhythms, then measure their consistency over time using the inverse of the Earth mover’s distance. Linear mixed-effects models were employed to compare movement rhythm variability among subpopulations.

**Results:**

During the pandemic, our cohort exhibited a shift in exercise routines, with a decrease in nonwalking physical exercises, while walking remained unchanged. Migrants and those living alone had less consistent daily movement rhythms compared to others. Those preferring on-site work maintained more consistent daily movement rhythms. Men and migrants returned to work more quickly after pandemic restriction measures were eased.

**Discussion:**

Our findings quantitatively show the pandemic’s unequal impact on different subpopulations. This study opens new research avenues to explore why certain groups return to on-site work, exercise levels, or daily movement rhythms more slowly compared to prepandemic times.

**Conclusions:**

Considering the pandemic’s unequal impact on subpopulations, organizations and policymakers should address diverse needs and offer tailored support during future crises.

## BACKGROUND AND SIGNIFICANCE

The COVID-19 pandemic imposed unprecedented levels of constraints on daily lives, while disproportionately affecting life rhythms and the well-being of different subgroups of individuals.^1^^,^^2^ Numerous studies have identified a decline in physical activity levels,^3^^,^^4^ deterioration of mental health^5^^,^^6^ and well-being,^7–9^ changes of sleep patterns, and a significant increase in digital media usage.^10^ These adverse impacts have been felt to a higher degree by different groups and communities, such as women,^11^^,^^12^ LGBTQ,^13^^,^^14^ and migrant populations.^15^^,^^16^ For example, women are shown to have experienced higher levels of stress and anxiety,^17^ more reductions in mobility,^18^ as well as a higher load in childcare^19^^,^^20^ during the pandemic. Similarly, migrant populations are reported to have experienced more stress due to lower levels of social support.^21^ Many studies explore the overall impact of restriction policies on different subgroups, but little is known about their effects on individuals’ daily rhythms and how life routines have changed.

Daily rhythms of activities and their consistency over time play an important role in people’s lives, both in terms of fulfilling the roles they play in society as well as at the individual level (eg, their mental and physical well-being).^22^^,^^23^ It is, therefore, crucial to understand how policies during the pandemic have led to changes in the daily rhythms of individuals. We analyze the daily rhythms of movement, ie, the daily movement activity patterns of people over 1 year during the COVID-19 pandemic. Specifically, we study how people distribute their movements throughout the course of the day, using digital records from fitness trackers combined with questionnaires answered by individuals. We explore the dynamics between daily rhythms of movement and on-site work attendance, ie, remote versus nonremote work. Furthermore, we investigate which socio-demographic factors are associated with maintaining higher levels of movement consistency and higher rates of returning to on-site work (nonremote work) throughout the course of our study.

Traditionally pen-and-paper (and later digital) surveys have been used to measure individuals’ daily activity rhythms and routines.^24^^,^^25^ Surveys can effectively reveal general routines, but they struggle to accurately capture daily activity rhythms and changes due to compliance issues and memory biases when conducted later. In recent years, the ubiquity of handheld and wearable devices^26^ has gradually mitigated this compliance and memory bias issue. Personal devices leave behind a massive volume of digital traces which contain granular information about users’ daily activity patterns and behaviors. Passive sensing using these devices allows capturing a person’s daily rhythms *in situ*, which minimizes recall bias from retrospective measurements and can be used over long periods at a time due to minimal extra effort for the user.

Before the COVID-19 pandemic, different studies have investigated daily rhythms of activity, for example, in social interactions via calls,^27^ text messages,^28^ and emails,^29^ phone usage,^30^ sleep,^31^^,^^32^ or web browsing.^33^ These studies have shown that, despite differences between individuals, people tend to keep consistent daily rhythms and allocate a similar portion of a certain activity (eg, making phone calls) to each section of the day (eg, morning, evening). Another type of activity that plays an important role in daily life is movement. Movement is a goal-directed behavior in which a specific type of movement is executed to attain a particular goal.^34^ Due to the constraints that the pandemic globally imposed, most working adults’ daily rhythms of movement were changed after the onset of the COVID-19 pandemic. Restrictions and individual circumstances led to diverse movement pattern changes during this period. Notably, daily rhythms of movement varied between regular commuters and remote workers during the pandemic.^3^ To the best of our knowledge, no research has yet quantified the daily rhythms of movement and their consistency over time during the pandemic.

## OBJECTIVE

Our study investigates how life routines changed during the pandemic and how these changes differ across socio-demographic factors like gender, age, and migrant status. We also aim to determine if routines have reverted to prepandemic norms. We collected fitness tracker records and questionnaire data from 128 working adults over a year (June 2021–June 2022). Questionnaire data showed shifts in physical activity from prepandemic levels. We created a metric to measure daily movement rhythms and consistency using step count data. Linear mixed-effects model (LMM) was used to analyze socio-demographic factors affecting movement consistency, as well as examine the relationship between this consistency metric and on-site work attendance versus remote work.

We observe a shift in exercise routines throughout the pandemic, evidenced by the significant decrease in average weekly time spent on physical exercises (other than walking) as opposed to the unchanged amount of time dedicated to walking. Secondly, we find that our participants are gradually coming back to work on-site with higher rates observed in male and migrant participants. Thirdly, our models confirm higher variability in daily rhythms of movement among migrant participants and those who live alone, as opposed to their counterparts. Fourthly, we observe a correlation between daily movement patterns and on-site work, where those who prefer working from home showed more varied daily movements. In summary, our study demonstrates the gradual, disproportional changes in various life routines and daily rhythms of movement throughout the pandemic, as well as the dynamics of these changes.

## MATERIALS AND METHODS

### Study design and data description

We use 2 different datasets: the cor:ona (comparison of rhythms: old vs. new) dataset, which we collected to address the research questions in this work, and the Oxford COVID-19 Government Response Tracker (OxCGRT)^35^ which is a publicly available dataset.

#### The cor:ona study

In June 2021, 128 full-time employees from a university in Finland were recruited. The main goal of this study was to examine the daily activity rhythms during different stages of the pandemic and to investigate how life routines change over time. Participants were full-time university employees without imminent workplace changes. They could withdraw from the study if their situation changed or for any other reason at any time. On an average, 3.2 participants left the study per month. Ninety-three participants remained in the study for the whole year. On an average, the participants took part for 253.5 days. Those who remained for more than 6 months received a discount code from Polar which was valid for purchasing similar fitness trackers. The participants were not compensated in any other way and were also informed that this study does not affect their employment in any way. The socio-demographic characteristics of the participants are presented in Table 1. For the question “Where are you from?” they were given 3 choices: Finland, Europe (except Finland), or outside of Europe. We refer to those who indicated that they are from Finland as “nonmigrant” and others as “migrant.” We did not ask about background or citizenship; the categorization is based solely on their response to this question. This study was approved by the Aalto University Research Ethics Committee.

**Table 1.** Socio-demographic characteristics of the cor:ona study participants

Attributes	Statistics
Gender, *N* (%)	
Female	85 (66.4%)
Male	42 (32.8%)
Nonbinary	1 (0.8%)
Role at university, *N* (%)	
Academic staff	56 (43.8%)
Service staff	72 (56.2%)
Age, *N* (%)	
25–35	60 (46.5%)
36–50	47 (37.0%)
51–66	21 (16.5%)
Origin, *N* (%)	
Finland	91 (70.9%)
Europe except Finland	15 (11.8%)
Outside of Europe	22 (17.3%)

##### Questionnaires

The participants completed a baseline questionnaire upon entering the study.

This included questions about socio-demographic information and life routines. The baseline questionnaire was filled out approximately 15 months after the start of the pandemic (mid-March 2020). Participants were asked to compare their activities in those 15 months (referred to in our study as the “early stage of the pandemic”) with the 15 months prior to the pandemic (referred to as “prior to the pandemic”). A short version of the baseline questionnaire was given to participants monthly. On an average, 103.5 participants answered the questionnaires each month. The participants completed an exit survey at the end of the study to assess their life routines during the year-long study period since they filled out the baseline questionnaire (referred to as the “late stage of the pandemic”).

##### Wearables

**Figure 1.**
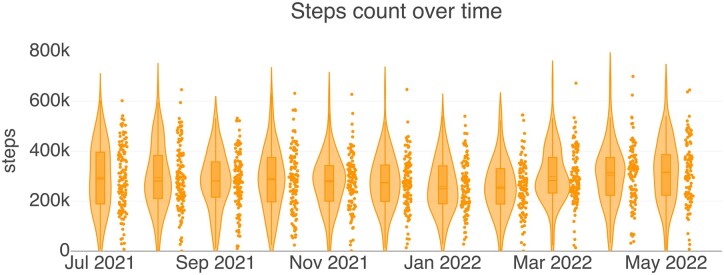
Step count values of all participants over the course of the study.

Participants were loaned new Polar Ignite fitness trackers (*N* = 121). Seven participants used their personal Polar fitness trackers. With users’ consent, we collected their personal data from Polar’s platform using Polar Accesslink API.^36^ This includes the step counts for each person in the study with hourly time resolution. In total, our dataset consists of 810 111 data points of step count and 48 228 data points from survey answers. Figure 1 shows the monthly step count of all participants over the course of the study.

#### Oxford COVID-19 Government Response Tracker

The Oxford COVID-19 Government Response Tracker (OxCGRT) captures government policies related to closures and containment in over 180 countries.^35^ The project evaluates national and subnational restriction policies and provides a list of composite indices measuring their extent. The sum of these indices is called the stringency index, which is a number between 0 and 100 (higher values indicating stricter policies) and is provided for each day during the pandemic for each region. We use the stringency index for Finland to assess the connection between the policies and movement/on-site work attendance.

### Measuring time allocation across activities

To assess changes in exercise and work routines during the pandemic, baseline and exit questionnaires asked about walking and nonwalking exercises, while monthly questionnaires inquired about the percentage of on-site work time in the past month. The detailed questions are presented in Supplementary Appendix SB.

### Quantifying daily rhythms of movement and their consistency over time

#### Daily rhythms of movement

**Figure 2.**
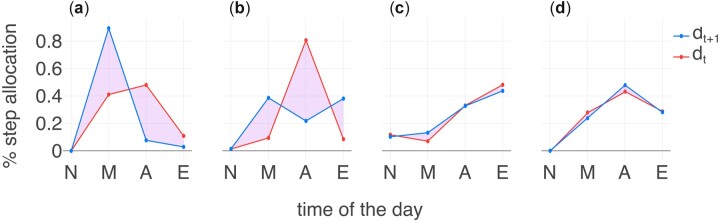
Visualization of movement consistency. A sample of 4 individual-level daily step allocations to demonstrate the level of movement consistency over time. The red line illustrates the step count distribution of *d_t_* and the blue line illustrates the distribution of *d_t_* _+1_. The markers indicate the distribution of steps in a single time segment (N: night midnight–6 am, M: morning 6 am–noon, A: afternoon noon–6 pm, E: evening 6 pm–midnight). The purple area indicates the distribution dissimilarity between 2 consecutive days. In panels (A) and (B), this area is considerably larger than that of panels (C) and (D), highlighting higher variability in day-to-day rhythms of movement.

To quantify the daily rhythms of movement, we calculate the temporal distribution of the daily step counts for each participant. We aggregate step counts at hourly resolution into larger time segments and compute the number of steps for each person and each day in the 4 different time segments: midnight—6am (night), 6am—noon (morning), noon—6pm (afternoon), and 6pm—midnight (evening). Step counts are normalized by dividing them by the total steps taken that day, allowing us to evaluate the proportion of steps taken during each time segment on a given day. The computation process is displayed in Figure 2.

Due to the inherent differences in movement rhythms between workdays and weekends, we consider them separately. Saturday and Sunday, are referred to as “weekends” and other days as “workdays”. We denote the distribution of step counts of the day *t* as *d_t_*, which represents the daily rhythms of movement. The selection of 4 time segments per day and their timing is informed by previous research in quantifying activity rhythms.^27^ We also evaluated the impact of different time-segmenting strategies on the consistency metrics (see Supplementary Appendix SC). The results were compared across various segmenting strategies and we found a consistent pattern in all cases, indicating that our method is robust to the granularity of time segmentation. Additionally, we used the Jensen-Shannon divergence as another distance metric to evaluate movement rhythms. When comparing these results with those obtained using the Earth Mover’s Distance (EMD), we found a moderately strong correlation between the 2 methods (see Supplementary Appendix SE).

#### Short-term movement consistency

The daily rhythms of movement can vary both in the short- and long term. We propose a metric called short-term movement consistency (*c_s_*) to measure how the daily rhythms of movement of a person vary from one day to the next. We quantify the movement consistency as the inverse of the EMD^37^ of the distributions of daily rhythms of movement between 2 consecutive days:


$$c_{t}^{s}=1/D\left(d_{t},d_{t+1}\right)$$

**Figure 3.**
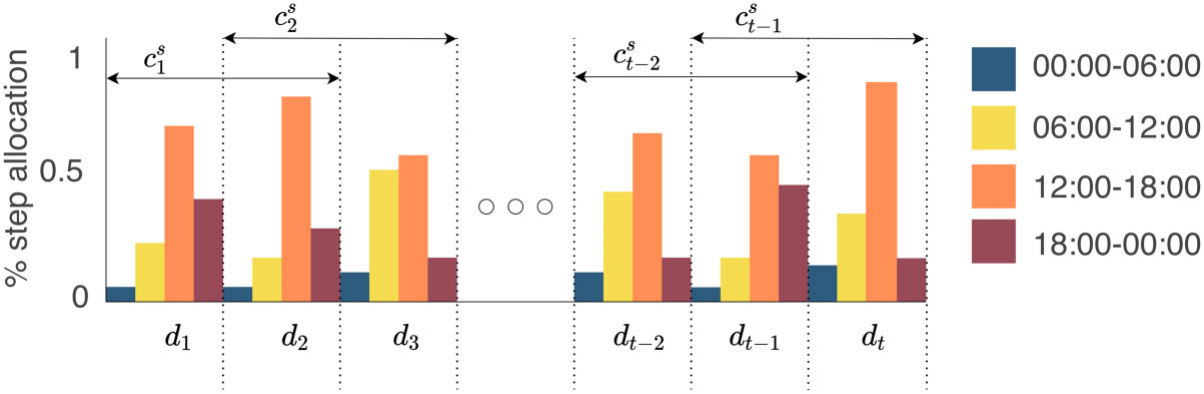
Short-term movement consistency computation. The short-term movement consistency is denoted as $c_{t}^{s}=1/D\left(d_{t},d_{t+1}\right)$and quantified as the inverse of the distance in step count distribution between *d_t_* and *d_t_* _+1_ of an individual.

The smaller the distance between each pair of daily rhythms of movement is, the more consistent the daily rhythms have been. Figure 3 depicts how short-term movement consistency is calculated.

To assess the changes in movement consistency over time, we calculate the average short-term movement consistency for each participant for each calendar month. For each month, the average short-term metric is defined as:


$$\bar{c_{\text{wd}}^{s}}=\sum_{t\in \hat{N_{\text{wd}}}}c_{t}^{s}/\left|\hat{N_{\text{wd}}}\right|,\;$$

where $\hat{N_{\text{wd}}}$ is the set of workdays within that month excluding Fridays. For weekends the average is defined as:


$$\bar{c_{\text{we}}^{s}}=\sum_{t\in \hat{N_{\text{we}}}}c_{t}^{s}/\left|\hat{N_{\text{we}}}\right|,\;$$

where $\hat{N_{\text{we}}}$ is the set of Saturdays within that month. So in this case, each $c_{t}^{s}$ is calculated by comparing the daily rhythm of a Saturday to the day after (Sunday).

#### Monthly- and long-term movement consistency

Short-term movement consistency is sensitive to changes in daily rhythms that happen on one day but fails to capture minor day-to-day changes following long-term trends due to factors like seasons or work-life. To capture these long-term, we propose 2 metrics: monthly movement consistency and long-term movement consistency, which measure the difference between an individual’s 1-day movement rhythm and their average (baseline) rhythm. The baseline, *d*, can be calculated based on the average movement daily rhythms of a person over an extended period of time. We denote the monthly baseline as *d_m_* and the long-term baseline, which consists of the whole time a person has participated in the study, as *d_l_*. We define monthly movement consistency, *c_m_*, as the inverse of the distance between the daily rhythms of movement *d_t_* and the monthly baseline $\overset{-}{d^{m}}$,


$$c_{t}^{m}=1/D\left(d_{t},\overset{-}{d^{m}}\right)$$

Similarly, we define long-term movement consistency as:


$$c_{t}^{l}=1/D\left(d_{t},\overset{-}{d^{l}}\right)$$

**Figure 4.**
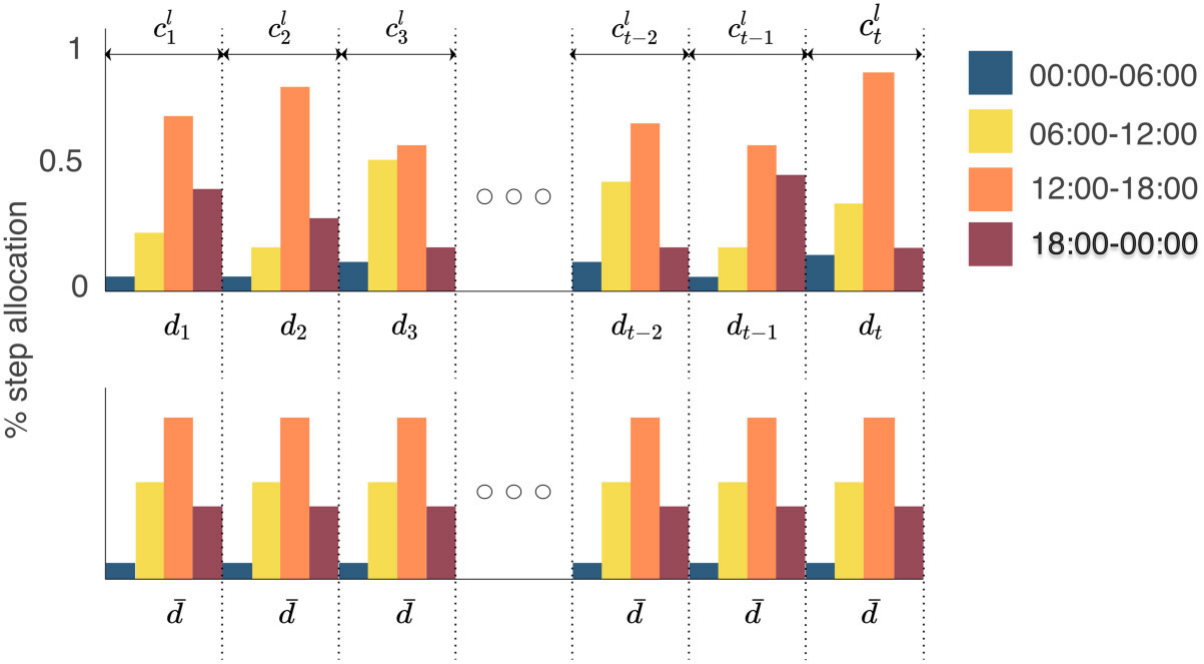
Long-term movement consistency calculation. The long-term movement consistency is denoted as $c_{t}^{l}=1/D\left(d_{t},\overset{-}{d^{l}}\right)$ and quantified as the inverse of the distance in step count distribution between *d_t_* and the average distribution *d_l_* of an individual.

Figure 4 depicts a schematic visualization of the long-term movement consistency.

Both these calculations will lead to one value for movement consistency for each day and each participant. Similar to short-term movement consistency we compute means of these values for weekdays and weekends separately, ie, we define:


$$\bar{c_{\text{wd}}^{m}}=\sum_{t\in N_{\text{wd}}}c_{t}^{m}/\left|N_{\text{wd}}\right|,$$

$$\overset{-}{c_{\text{we}}^{m}}=\sum_{t\in N_{\text{we}}}c_{t}^{m}/\left|N_{\text{we}}\right|,$$

where *N*_wd_ is the set of workdays and *N*_we_ is the set of days in weekends within a month. $\overset{-}{c_{\text{wd}}^{l}}$ and $\overset{-}{c_{\text{we}}^{l}}$ are calculated similarly to $\overset{-}{c_{\text{wd}}^{m}}$ and $\overset{-}{c_{\text{we}}^{m}}$, by replacing $c_{t}^{m}$ with $c_{t}^{l}$ in the 2 formulas above respectively.

### Models: notations and selection

We propose 3 models to evaluate the relationship between movement consistency and socio-demographic factors. We consider 3 different consistency metrics as the dependent variable (DV): short-term weekday (Model 1a), long-term weekday (Model 1b), and long-term weekend (Model 2). We do not consider short-term weekend metric since it is just the difference between Saturday and Sunday and long-term data is needed for this comparison. We take into account the following independent variables (IVs): age, gender, origin, cohabitation status (whether the participant lives with someone else), children status (whether the participant has children), and role at the university (academic staff or service staff). Additionally, we consider a 2-way interaction between gender and children status to account for the role of gender in childcare duty.

We build Model 3 to evaluate the relationship between on-site work attendance (reported in the monthly surveys as the percentage of on-site work time in the past calendar month) and movement consistency. To analyze the association between movement consistency and on-site work attendance, in addition to the above IVs, we also include the average long-term workday consistency metric calculated by getting the mean of all workday consistencies within the month. All the above response variables are measured at monthly intervals. Models 1a, 1b, and 2 use the monthly average of the short- and long-term movement consistencies respectively for each month.

To control for repeated measurements, we employ a linear mixed-effects model (LMM).^38^ Models 1a, 1b, and 2 include participants as random effects to account for the intraindividual difference. For Model 3, we incorporate calendar months (July, August, September, etc.) as random effects to account for the variations in on-site work rates due to restrictions policies. Model 3 does not include individual-specific random effects due to insufficient data for incorporating both levels of random effects. Instead, individual-level variation is captured through extensive fixed effect covariates. For individual *i*, at calendar month *j* ∈ {7(July),8(August),.,5(May)}, we denote *Y_ij_* as the variable of interest and *x_ij_* as the covariate. The intercept for the random effect is denoted as *u_j_*. We formally describe the models as follows:

Model 1a, 1b, 2: *Y_ij_*=β_0_+β_1_*x_ij_*_1_+β_2_*x_ij_*_2_+…+β_7_*x_ij_*_7_+*u_j_*+ϵ_*ij*_,

Model 3: *Y_ij_*=β_0_+β_1_*x_ij_*_1_+β_2_*x_ij_*_2_+…+β_7_*x_ij_*_7_+β_8_*x_ij_*_8_+*u_j_*+ϵ_*ij*_,

where the IVs of individual *i* during month *j* are:

*x_ij_*_1_=age group, *x_ij_*_2_=gender, *x_ij_*_3_=origin,

*x_ij_*_4_=live with someone, *x_ij_*_5_=have children,

*x_ij_*_6_=gender×live with someone, *x_ij_*_7_=gender×have children,

*x_ij_*_8_=monthly average of long-term workday movement consistency.

The *P*-values and 95% confidence intervals for the estimates of coefficients are calculated using a parametric bootstrap. We perform variance inflation factor (VIF) analysis for all models. The max GVIF for any predictor is 2.2 (gender×have children), which is under the recommended threshold.^39^

#### Data exclusion and preprocessing

We exclude participants with less than 20% available step count data during the 1-year study, leaving 111 participants. Days without step count data are omitted. To account for missing data from dropouts or infrequent device use, we exclude participants without step count data for 5 workdays or 2 full weekends per month. Nonbinary participants are excluded from gender-related analysis for privacy, and those with over 7 days of leave per month (asked monthly through the questionnaire) are excluded from on-site work attendance analysis (Model 3). All numerical variables were standardized in all models.

## RESULTS

### Changes in daily activities after the onset of the pandemic

Amid the COVID-19 pandemic, lockdowns and gym restrictions induced a shift from on-site work to remote work and traditional exercises to alternative exercise forms. For instance, at our study’s university, employees were encouraged to hold remote meetings during outdoor walks. We examine shifts in work location and exercise types using participant questionnaires responses.

In Table 2, we show the reported estimate of time allocation for on-site work and exercises in 3 periods: pre-, early, and late stages of the pandemic. The average hours for walking weekly do not differ significantly between stages. Nonwalking exercise hours significantly drop during early (mean = 2.58 h, SD = 2.51 h) and late stages (mean = 2.76 h, SD = 2.38 h) compared to the prepandemic level (mean = 3.62 h, SD = 2.75 h). This decrease is significant for female and nonmigrant participants but not for male and migrant participants. The monthly percentage of time spent on-site drops significantly from the prepandemic (mean = 83.64%, SD = 22.67%) to the early stage (mean = 9.94%, SD = 19.39%) and partially recovers in the late stage (mean = 32.56%, SD = 25.16%).

**Table 2.** Average amounts of activities at different stages of the pandemic compared to the prepandemic time

Attributes	Pandemic		
	Pre	Early	Late
Avg. hours per week for walking			
All	5.33	4.83	5.97
Male	4.53	3.67	4.93
Female	5.68	5.43	6.61
Nonmigrant	5.37	5.17	6.07
Migrant	5.33	4.18	6.00
Avg. hours per week for nonwalking exercises			
All	3.62	2.58***	2.76**
Male	3.71	3.37	3.04
Female	3.54	2.21***	2.57**
Nonmigrant	3.42	2.15***	2.45**
Migrant	4.03	3.51	3.33
% of working time spent on-site			
All	83.64	9.94***	32.56***
Male	83.42	13.57***	43.27***
Female	83.75	8.20***	27.44***
Nonmigrant	82.38	8.62***	27.87***
Migrant	86.54	12.97***	43.32***

*Note*: Weekly walking, nonwalking exercise, and the percentage of on-site work time are compared across prepandemic, early, and late pandemic stages. The late stage on-site work percentage is based on participants who completed at least 5 monthly surveys. Prepandemic stage serves as a reference, comparing means from other stages for specific activities and subpopulations. All comparisons are made using Wilcoxon signed rank test. Asterisks denote the significance of the results.

** *P* < .01.

*** *P* < .001.

Compared to the early stage, there is an increase in the trend for walking (*U* = 1333.0, *P* = .02) and on-site work attendance (*U* = 168.0, *P* < .001) during the late stage of the pandemic, as individuals gradually return to pre pandemic levels. However, nonwalking exercises remains at the same level (*U* = 1210.0, *P* = .14), implying that walking might compensate for other exercise types in daily routines.

**Figure 5.**
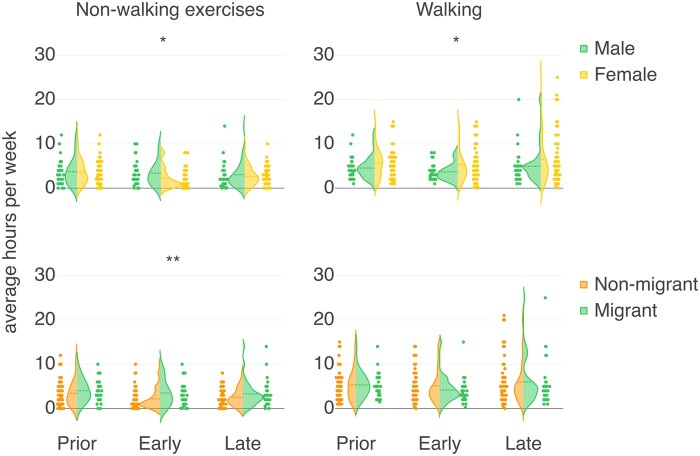
Comparison of time allocation for different activities among subpopulations during each stage of the pandemic using Mann-Whitney *U* test. Asterisks denote the significance of the comparison in the corresponding stage. *P < .05, **P < .01, ***P < .001.

Distributions of nonwalking and walking exercises and subpopulation comparisons are shown in Figure 5. There is no significant difference in the time spent on walking between males and females, neither during the prepandemic stage (*U* = 790.0, *P* = .06) nor the late stage (*U* = 842.5, *P* = .12). However, during the early stage of the pandemic, females show a significantly higher amount of time spent walking (*U* = 727.5, *P* = .02). Conversely, in regards to nonwalking exercises, females tend to spend significantly less time during the early stage of the pandemic compared to males (*U* = 709.5, *P* = .01), while no significant differences are found in the prepandemic (*U* = 989.0, *P* = .49) and the late stage (*U* = 978.5, *P* = .46).

#### Movement consistency varies among different subpopulations

**Figure 6.**
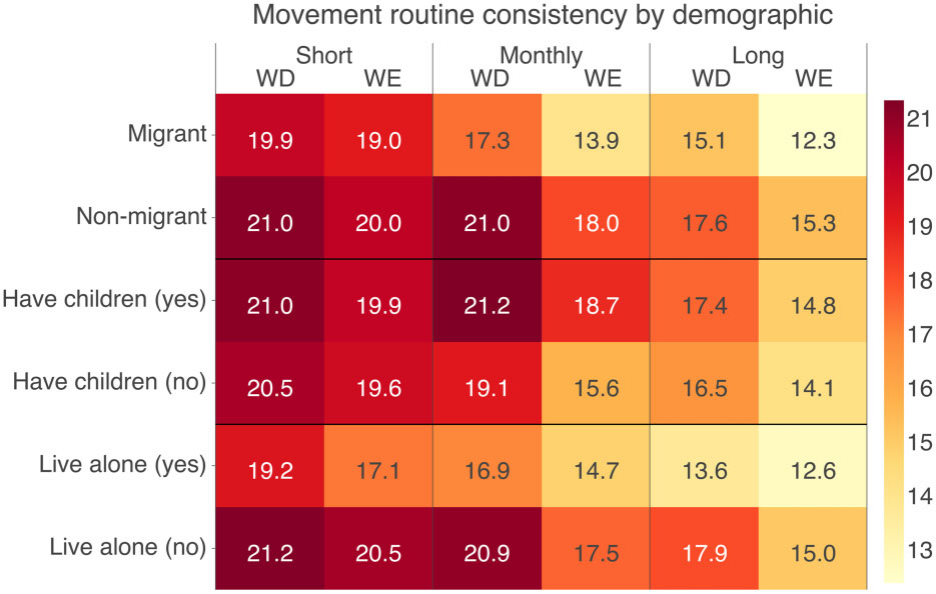
Movement consistency variability by socio-demographic factors. Lower values indicate lower consistency. Weekday is denoted as WD and weekend is denoted as WE. The monthly consistency is measured as the inverse of the distance between the 1-day distribution against the monthly average distribution. For all groups, weekend variability is generally higher than variability of workdays. Migrant participants tend to have the lowest consistency level.

Individuals may have different variability in daily rhythms of movement due to societal responsibilities, such as parenting, or the nature of their occupation. Figure 6 shows the variations in movement consistency among subpopulations, with a greater contrast observed in long-term daily rhythms of movement. We examine these differences by comparing the daily rhythms of movement of all participants while controlling for socio-demographic factors. Table 3 shows the results of Model 1a and 1b (short-term and long-term weekend movement consistency). Both models suggest that participants living alone tend to have lower short-term (β=−0.33, *P* = .022) and long-term (β=−0.78, *P* < .001) movement consistency. Similarly, migrants have lower long-term movement consistency (β=−0.61, *P* = .004) but the difference in short-term movement consistency is not significant (β=−0.19, *P* = .210). The result for Model 2 (long-term weekend consistency) is presented in Supplementary Appendix SA.

**Table 3.** Results for socio-demographic variables predicting short- and long-term workday consistency

	Model 1a (short term)		Model 1b (long term)	
	Est.	95% CI	Est.	95% CI
(Intercept)	0.22	−0.08 - 0.53	0.48	0.06 - 0.91*
Role (service staff)	−0.08	−0.36 - 0.21	−0.17	−0.56 - 0.22
Gender (male)	−0.16	−0.45 - 0.15	−0.11	−0.30 - 0.08
Live alone (yes)	−0.33	−0.61 - −0.05*	−0.78	−1.15 - −0.40***
Have children (yes)	−0.10	−0.40 - 0.19	−0.29	−0.70 - 0.11
Origin (migrant)	−0.19	0.50 - –0.10	−0.61	−1.01 - −0.20**
Age	0.01	−0.12 - 0.14	−0.02	−0.19 - 0.15
Have children (yes)×gender (male)	0.22	−0.26 - 0.70	0.31	−0.37 - 0.98
Random effects				
σ^2^	0.74		0.34	
τ_00participant_	0.25		0.61	
ICC	0.25		0.64	
*N*_participant_	111		111	
Observations	885		912	
Marginal *R*_2_/conditional *R*_2_	0.025/0.271		0.131/0.687	

*Note*: Asterisks denote the significance of the results.

* *P* < .05.

** *P* < .01.

*** *P* < .001.

### Movement consistency positively correlates with working on-site

Different subpopulations show varied rates of returning to on-site work, potentially linked to movement consistency. Higher on-site work rates likely correlate with more regular daily movement rhythms due to structured schedules. Model 3 confirms a positive correlation between long-term movement consistency and on-site work attendance, as one unit increase in movement consistency results in a 7%±2% (95% CI) increase in time spent on-site. Among all demographic factors, male and migrant participants tend to spend 12%±6% and 13%±5% (95% CI) more time on-site on an average than their counterparts respectively. Individuals who live alone tend to spend more time at work on-site, with an increase of 13%±5% (95% CI) compared to those who live with others. The full results are displayed in Supplementary Appendix SA.

The connection between movement and on-site work may be influenced by restriction measures. Our theory is that tighter restrictions on public areas could result in more regular daily movement rhythms, due to fewer socializing chances. To verify this, we evaluate variability using Model 3’s random intercepts, which assess monthly on-site work inclination. When comparing this property against the stringency index (see “Oxford COVID-19 Government Response Tracker”), we observe a positive relationship: participants are willing to work from home when the policy is tightened (*r* = −0.897, *P* < .001) (see Supplementary Appendix SD). We retested Models 1a and 1b using calendar month as a random effect. Model 1b’s random intercepts indicate long-term movement consistency variability each month. Results reveal a negative correlation between variability and the stringency index (*r* = −0.836, *P* = .001), meaning stricter restrictions relate to less consistent daily movement rhythms. Overall, participants adhered to restrictions and had more diverse activities while working remotely.

## DISCUSSION

Our study analyzes a high-resolution, long-term dataset of working adults during the COVID-19 pandemic, focusing on: (1) time allocation comparison for daily activities across pandemic stages; (2) new methods to infer daily movement rhythms and consistency from fitness trackers; (3) variability in movement rhythms among subpopulations; and (4) the relationship between movement rhythms and on-site work attendance. We proposed a method using step count data to identify movement rhythm differences. After controlling for factors like age, gender, and workplace role, we found higher variability in movement consistency among migrants and those living alone, and a positive link between movement consistency and on-site work likelihood.

Walking levels stayed the same from pre- to early pandemic, and then rose from early to late pandemic. These changes could be attributed to a variety of factors, including common leisure activity^40^ or an effective coping mechanism against boredom.^41^ During the pandemic, travel behaviors changed significantly as private transport modes (walking, car) were preferred over public transport modes (bus, train) due to perceived risks of COVID-19 transmission.^42^ Moreover, commuting distances were shorter and the majority of trips were made for shopping purposes,^43^ which encouraged walking as a mode of transportation even more.

During the pandemic’s early stage, all participants, especially females, reduced their time spent on nonwalking exercises significantly. This contradicts studies showing a larger decrease in physical activity among males, attributed to differences in exercise habits and intensity.^44–46^ However, the gender difference in nonwalking exercises became insignificant during the late stage.

Individuals’ daily movement rhythms were consistent short-term, with higher variability in monthly and long-term measurements for migrants and those living alone. These changes might indicate attempts to combat loneliness through increased social interaction.^47^ The same subpopulations had higher on-site work rates, possibly to alleviate social isolation. During the March 2022 survey, a question was presented to the participants regarding their reasons for returning to on-site work. The findings revealed that 53% of migrant participants cited social interaction as their main motivation for returning to on-site work, compared to 35% of nonmigrant participants, emphasizing the importance of social interaction for those experiencing loneliness.

Males had a higher on-site work attendance than females with no significant effect from gender-specific childcare duties found, contrary to previous studies.^11^^,^^12^^,^^48^^,^^49^ Possible reasons include even distribution of childcare, Finland’s lack of a complete lockdown, school reopenings reducing gender disparity, and males’ higher risk-taking behavior.^50^^,^^51^ Studies show that men have a lower propensity to engage in health-protective behaviors and perceive less risk than women during the pandemic.^52^^,^^53^

In terms of methodology, a common approach in studies utilizing similar data sources (accelerometer-generated step count) is to use descriptive statistics to give a general sense of physical activity levels or how they differ between subpopulations.^54^^,^^55^ This approach is also employed in pandemic-related research, where studies contrast activities across multiple periods (eg, prepandemic vs postpandemic, prelockdown vs postlockdown).^56^^,^^57^ In our study, we use a combination of descriptive statistics and linear mixed-effects models, which is a frequently utilized method.^58^ This allows us to examine the differences in daily movement rhythms between demographic factors and explore the relationship between movement rhythms and workplace preference. In addition to that, we introduce a novel method to quantify the consistency of the daily movement rhythms over time. In the future, this method can be used for other datasets of step count data. However, the application of this method is not limited to step counts. This method can be used to derive daily rhythms for any quantifiable activity that occurs within predetermined time intervals throughout the day and to evaluate the consistency of the daily rhythms over time. To obtain a more comprehensive understanding of the consistency of daily rhythms for various types of activities that people engage in throughout the day, in future studies, we will investigate the association between the consistency of daily rhythms derived from different data streams from individuals that are collected simultaneously.

### Limitations

Our work is not without limitations. First, to measure step counts we use consumer-grade fitness trackers. Step counts are calculated based on Polar’s proprietary algorithms and we do not have access to unprocessed data. However, as our work primarily relies on relative step counts throughout the day, rather than absolute numbers, we do not expect to have a large bias due to this. Second, our study includes a modest sample size of 128 full-time employees at a Finnish university over a 1-year period. Thus, our results cannot be generalized to other societies, workplaces, or socio-demographics.

Furthermore, the limited timeframe of our study may not provide a comprehensive view of the pandemic’s long-term impacts on movement rhythms. Additionally, while our study is specifically designed to focus on the impacts of the late-stage COVID-19 pandemic on daily activity rhythms, it may overlook other influential factors on daily activity rhythms and routines, such as individual lifestyle choices, work-related stressors, and personal health factors among others. Lastly, due to the lack of prospective data collected prior to the pandemic, comparisons against prepandemic activities had to depend on self-reported measures, which may contain recall bias. Future mixed-method studies can apply our methodology to diverse demographic groups, quantifying daily movement rhythms and their temporal consistency, and exploring how various personal, workplace, or societal changes affect these rhythms.

## CONCLUSION

Our research expands upon prior studies on movement rhythms during the pandemic by utilizing a high-resolution, objective method for measuring movement rhythms, improving upon survey-based approaches. We suggest a straightforward approach to extract movement rhythms from fitness trackers, complementing existing techniques such as mapping smartphone locations^59^ and geo-located Twitter.^60^ Additionally, our dataset allows for capturing movement rhythms over an extended time, enhancing the reliability of the proposed method. Even though our findings cannot be directly applied to other demographics or crises, this study still offers valuable insight into an issue of significant importance in policy-making, especially for strategies aimed at mitigating adverse impacts on diverse social groups. These insights remain relevant in the postpandemic landscape as we readjust our daily routines, living, and working styles.

## Supplementary Material

ocad140_Supplementary_Data

## Data Availability

The data underlying this article will be shared on reasonable request to the corresponding author.
