# Supplementary material for: The impact of the COVID-19 pandemic on daily rhythms
Source: J Am Med Inform Assoc. 2023 Aug 7;30(12):1943–53. doi: 10.1093/jamia/ocad140 (PMC10654873; doi:10.1093/jamia/ocad140)
Supplement: ocad140_Supplementary_Data [file ocad140_supplementary_data.zip › ocad140_Supplementary_Data/appendix_re_2.docx]

**Appendix A Mixed model: Controlling for**

**demographic heterogeneity**

# Model 2: Weekend movement consistency

|  | Est. | 95% CI |
| --- | --- | --- |
| (Intercept) | 0.22 | -0.08 – 0.53 |
| Role (service staff) | -0.08 | -0.36 – 0.21 |
| Gender (male) | -0.16 | -0.45 – 0.15 |
| Live alone (yes) | -0.33 | -0.61 – -0.05* |
| Have children (yes) | -0.10 | -0.40 – 0.19 |
| Origin (migrant) | -0.19 | -0.50 – 0.10 |
| Age | 0.01 | -0.12 – 0.14 |
| Have children (yes) x Gender (male) | 0.22 | -0.26 – 0.70 |

**Table A1**: Results for socio-demographic variables predicting short and longterm workday consistency. Asterisks denote the significance of the results.

**p <* 0*.*05, ***p <* 0*.*01, ****p <* 0*.*001.

# Model 3: On-site work attendance

|  | Est. | 95% CI |
| --- | --- | --- |
| (Intercept) | 0.30 | 0.22 – 0.37*** |
| Long-term workday consistency | 0.07 | 0.04 – 0.09*** |
| Role (service staff) | -0.12 | -0.17 – -0.07*** |
| Gender (male) | 0.12 | 0.06 – 0.17** |
| Live alone (yes) | 0.08 | 0.03 - 0.13*** |
| Have children (yes) | -0.10 | -0.06 – 0.04 |
| Origin (migrant) | 0.13 | 0.08 – 0.18*** |
| Age | 0.02 | 0.00– 0.04* |
| Have children (yes) x Gender (male) | -0.01 | -0.10 – 0.07 |
| Random effects *σ*2 | 0.08 |  |
| *τ*^00^*month* | 0.01 |  |
| ICC | 0.08 |  |
| *Nmonth* | 11 |  |
| Observations | 912 |  |
| Marginal *R*^2^ / Conditional *R*^2^ | 0.151 / 0.223 |  |

**Table A2**: Results for socio-demographic variables and long-term workday consistency predicting on-site work attendance rate. Asterisks denote the significance of the results. **p <* 0*.*05, ***p <* 0*.*01, ****p <* 0*.*001.

**Appendix B Routine-related questions**

| Routine | Question |
| --- | --- |
|  | During the pandemic; what percentage of your working time did you spend on-site? (b) |
| Workplace | Prior to the pandemic; what percentage of your working time did you spend on-site? (b)  If you worked in (part of) *month* what percentage of your working time did you spend on-site ?  (do not answer if you did not work at all in *month*) (m) |
|  | Prior to the pandemic; how many hours per week on average did you walk? (b) |
| Walking | During the pandemic; how many hours per week on average have you walked? (b)  In the late stages of the pandemic; how many hours per week on average have you walked? (e) |
|  | Prior to the pandemic; how many hours per week on average did you engage in non-walking exercise (e.g., riding a bike; weightlifting; etc)? (b) |
| Non-walking exercise | During the pandemic; how many hours per week on average have you engaged in non-walking exercise  (e.g., riding a bike; weightlifting; etc)? (b)  In the late stage of the pandemic; how many hours per week on average have you engaged in non-walking exercise  (e.g., riding a bike; weightlifting; etc)? (e) |

**Table B3**: Questions about life routines. Questions marked with **(b)**, **(e)**,

**(m)** are asked in the baseline, exit, and monthly surveys, respectively.

**Appendix C Different segmenting strategies**

**for computing distribution of steps**


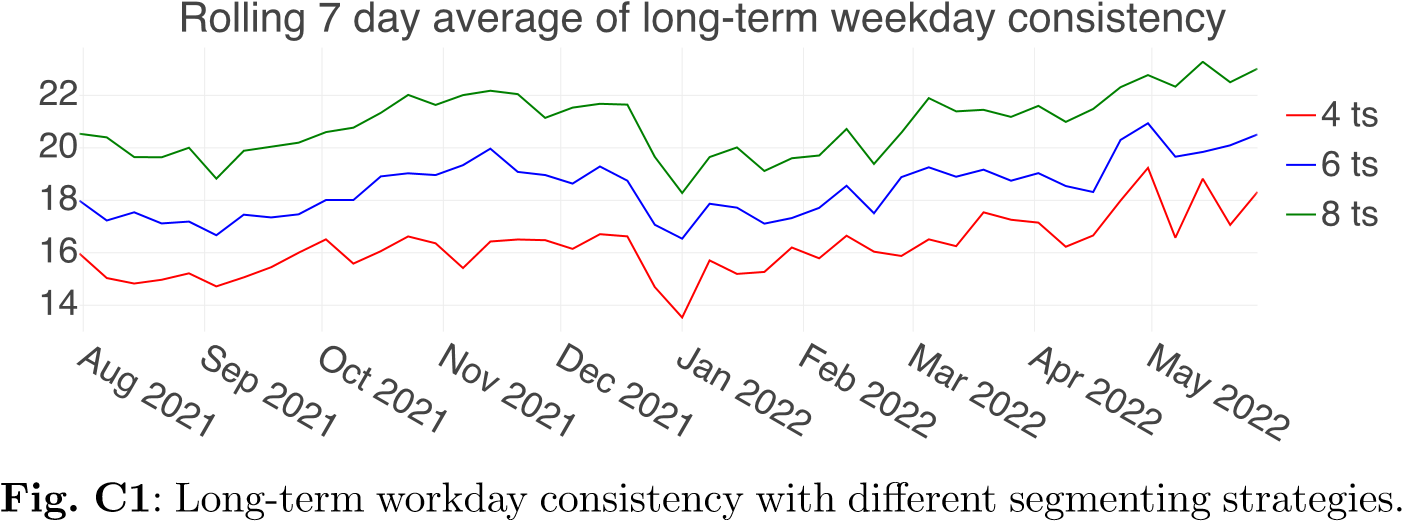


In Figure C1, we demonstrate long-term workday consistency with different time segmenting strategies. The trend is represented as a 7-day rolling average. We can observe that the smaller the time segment (ts), the less drastic the change is. However, the general temporal trend holds regardless of the number of time segments.

**Appendix D Relationship between long-term**

**movement rhythms consistency, onsite-work attendance, and stringency index**


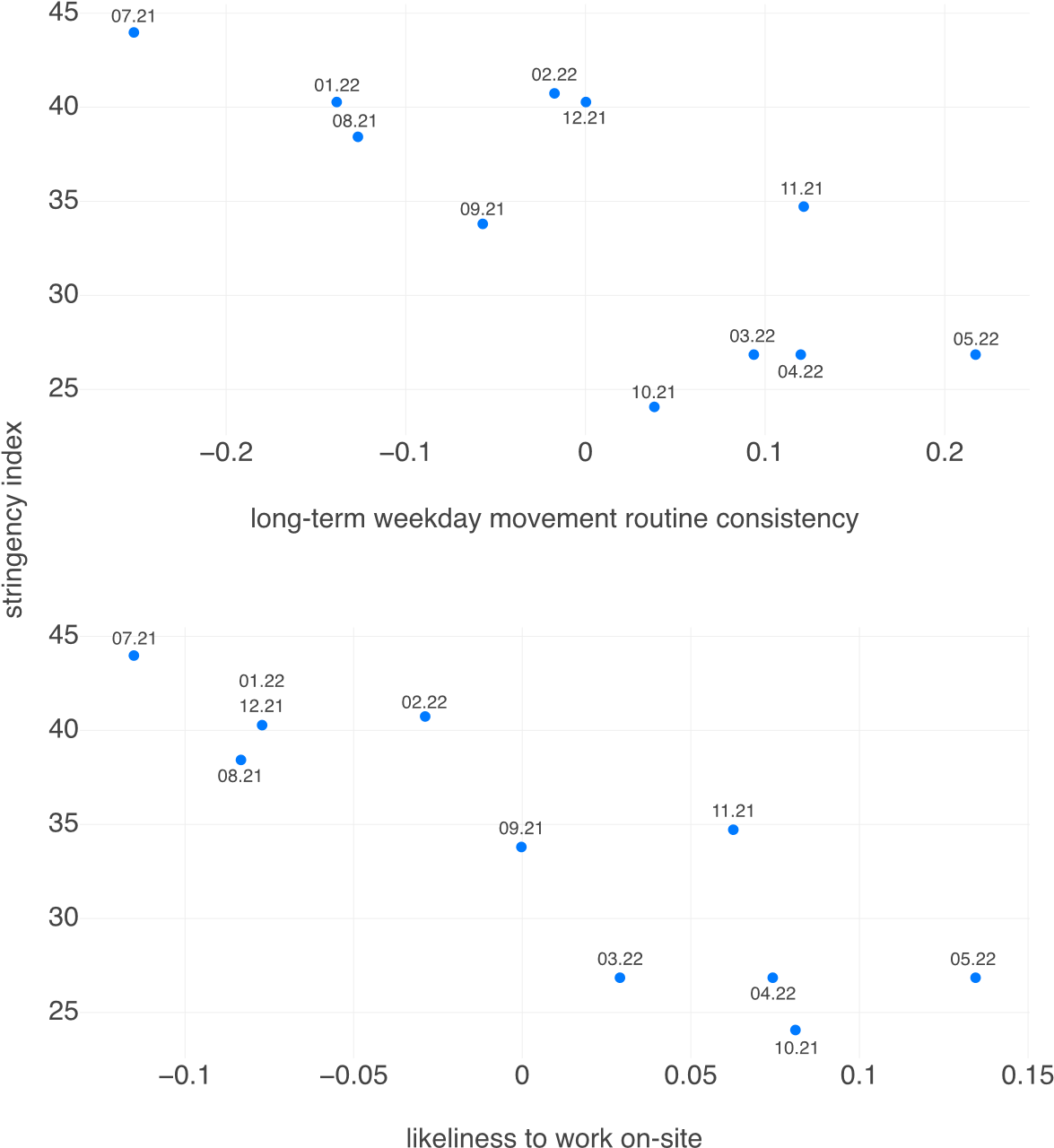


**Fig. D2**: Relationship between stringency index and movement consistency and likeliness to work on-site. The random intercepts from Model 1b indicate the variability of the long-term workday movement consistency and the random intercepts from Model 3 indicate the likeliness to work on-site.

**Appendix E Movement consistency calculated**

**using Jensen-Shannon divergence**

Model 1a (short-term)

Model 1b (long-term)

|  | Est. | 95% CI | Est. | 95% CI |
| --- | --- | --- | --- | --- |
| (Intercept) | 0.23 | -0.12 – 0.58 | 0.43 | 0.03 – 0.83* |
| Role (service staff) | -0.08 | -0.33 – 0.28 | -0.08 | -0.44 – 0.28 |
| Gender (male) | -0.17 | -0.51 – 0.23 | -0.30 | -0.71 – 0.11 |
| Live alone (yes) | -0.29 | -0.60 – 0.02 | -0.58 | -0.94 – -0.23*** |
| Have children (yes) | -0.14 | -0.47 – 0.21 | -0.07 | -0.46 – 0.32 |
| Origin (migrant) | -0.29 | -0.63 – 0.07 | -0.59 | -0.98 – -0.21** |
| Age | 0.11 | -0.01 – 0.24 | 0.07 | -0.06 – 0.20 |
| Have children (yes) x Gender (male) | 0.24 | -0.35 – 0.77 | 0.32 | -0.31 – 0.95 |

**Table E4**: Results for socio-demographic variables predicting short and longterm workday consistency calculated using Jensen-Shannon divergence metric.

Asterisks denote the significance of the results. **p <* 0*.*05, ***p <* 0*.*01, ****p <*

0*.*001.


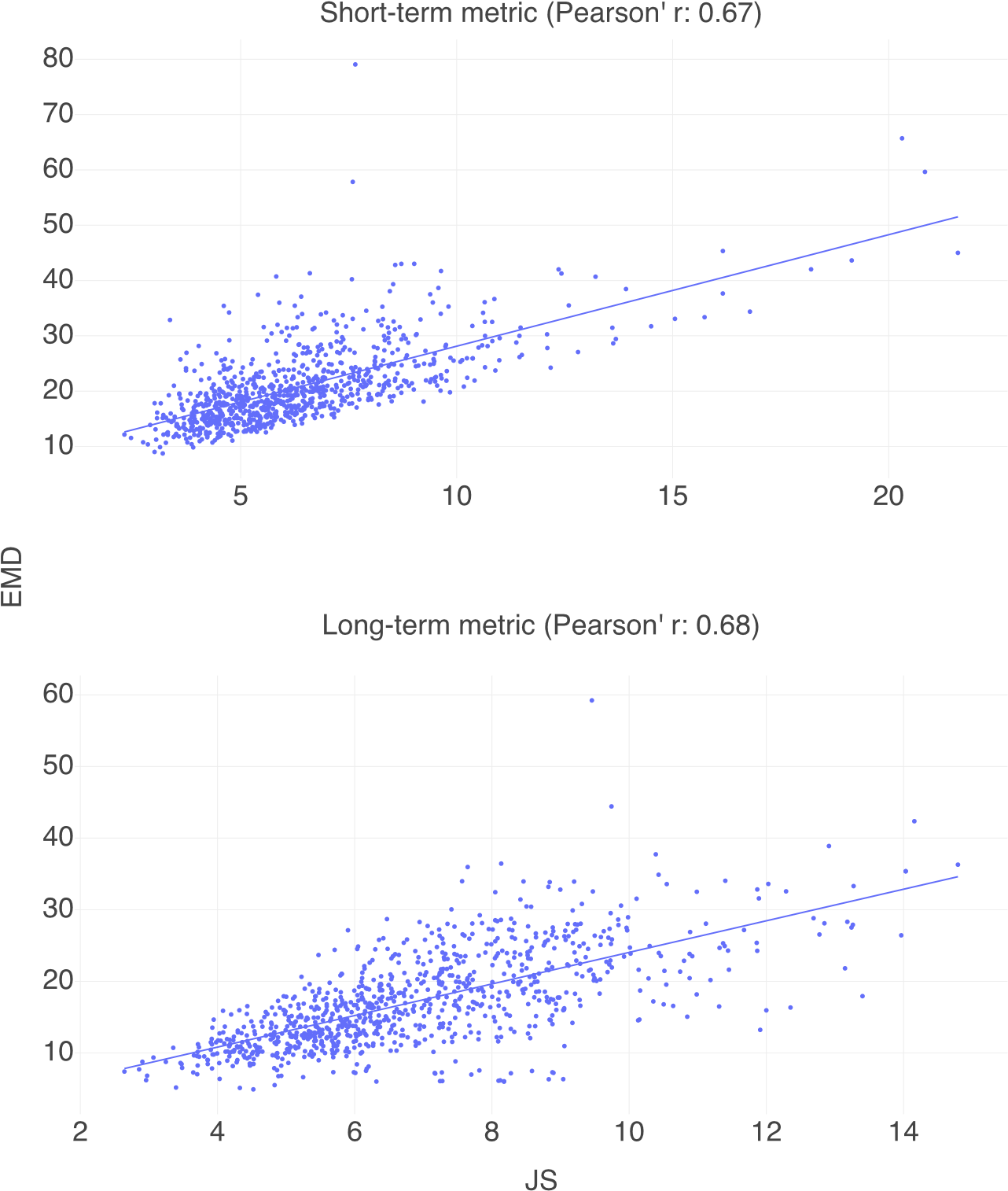


**Fig. E3**: Scatter plot of EMD and JS weekday movement rhythm
