# Supplementary figures and images for: The impact of the COVID-19 pandemic on daily rhythms
Source: J Am Med Inform Assoc. 2023 Aug 7;30(12):1943–53. doi: 10.1093/jamia/ocad140 (PMC10654873; doi:10.1093/jamia/ocad140)

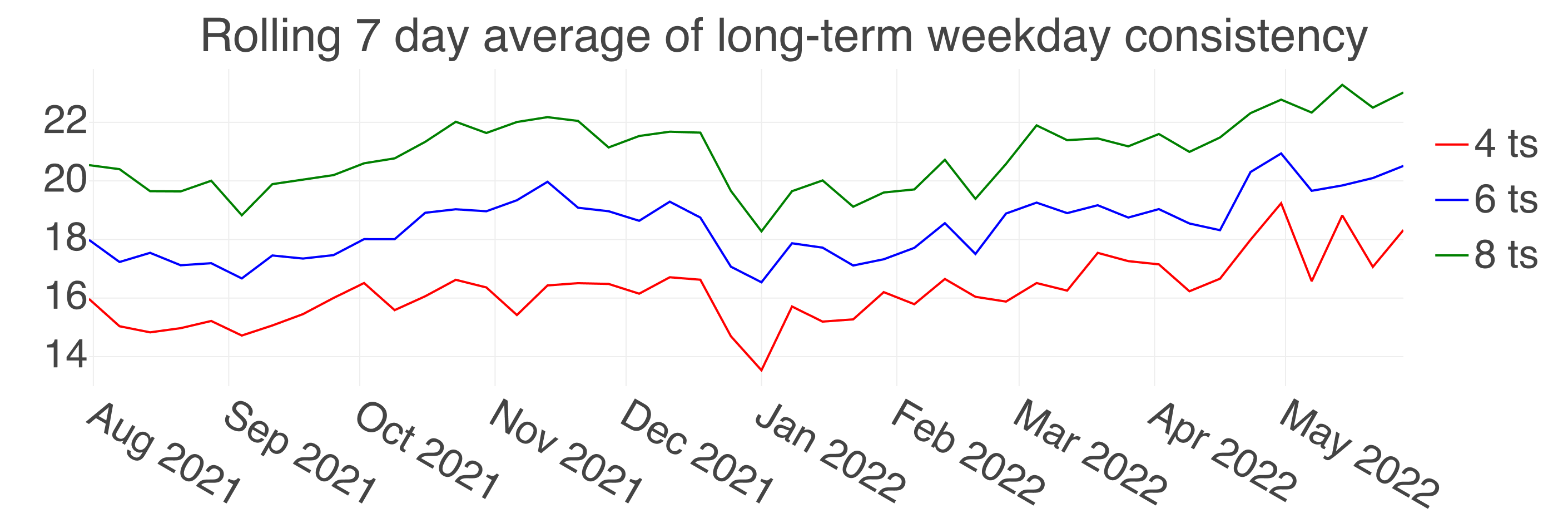

Supplement: ocad140_Supplementary_Data [file ocad140_supplementary_data.zip › ocad140_Supplementary_Data/appedix_fig_1_multiple_binned_consistency.png]

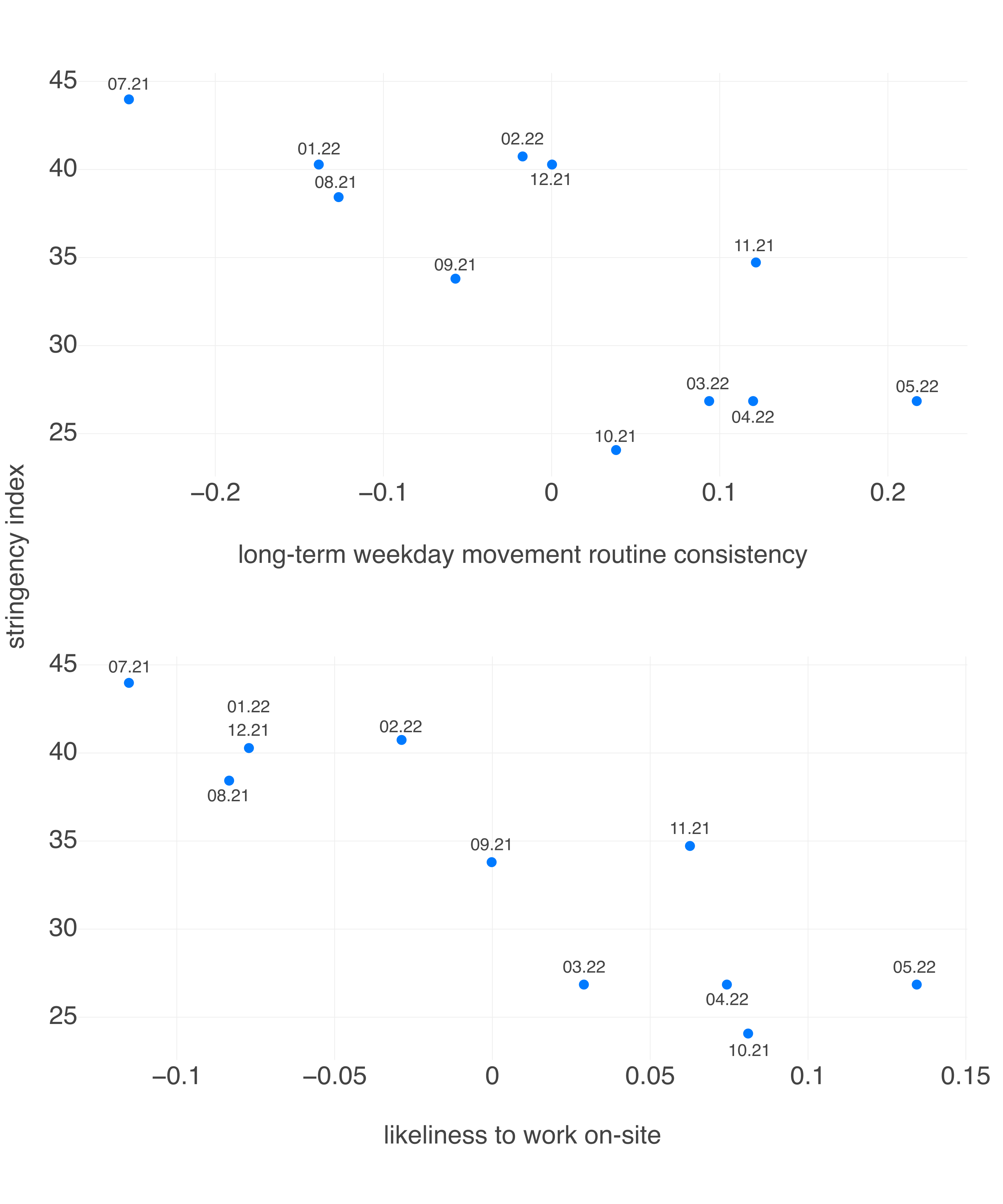

Supplement: ocad140_Supplementary_Data [file ocad140_supplementary_data.zip › ocad140_Supplementary_Data/appendix_fig_2_stringency_index_vs_ranef_long_workplace.png]

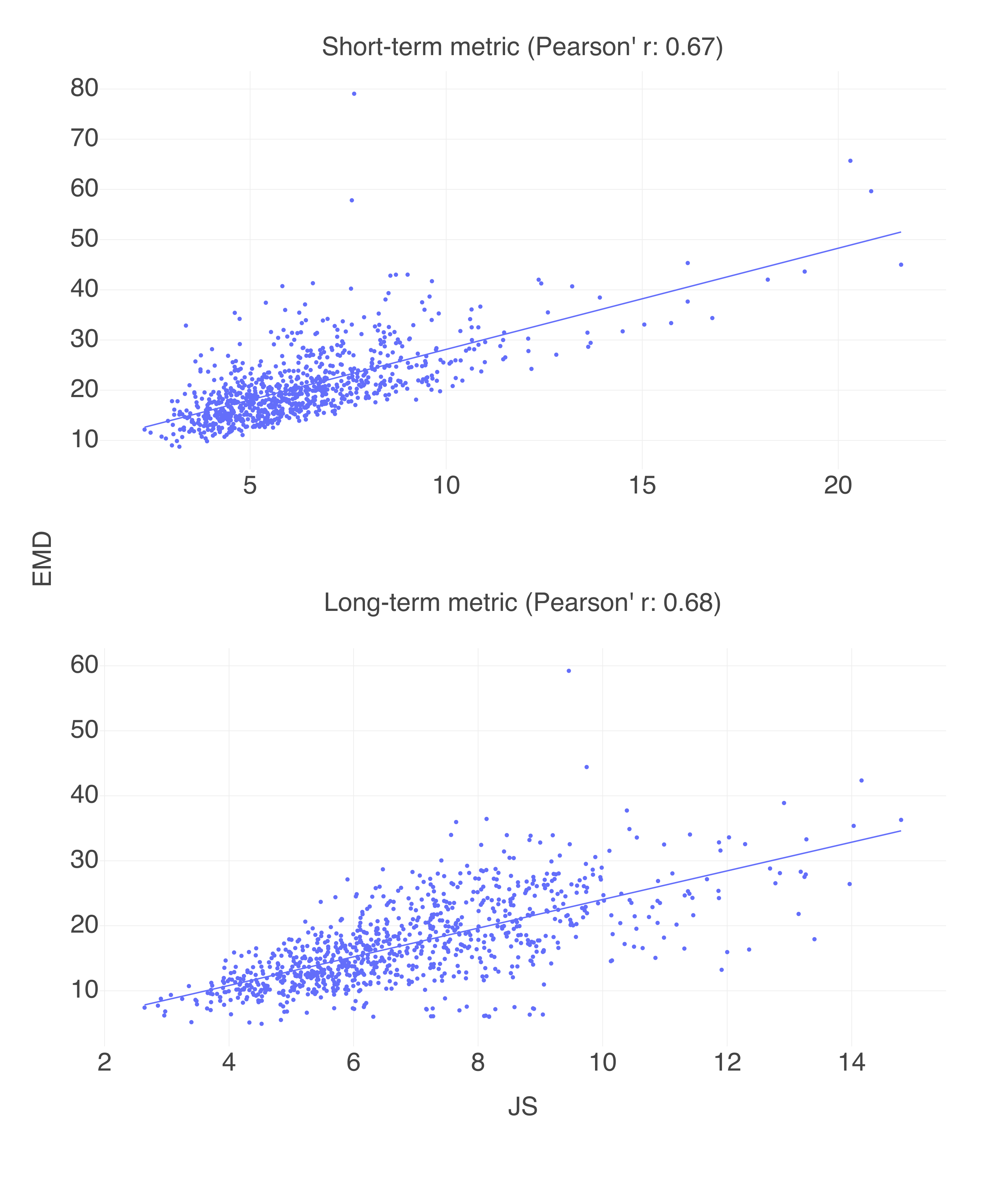

Supplement: ocad140_Supplementary_Data [file ocad140_supplementary_data.zip › ocad140_Supplementary_Data/appendix_fig_3_emd_js_long_short.png]

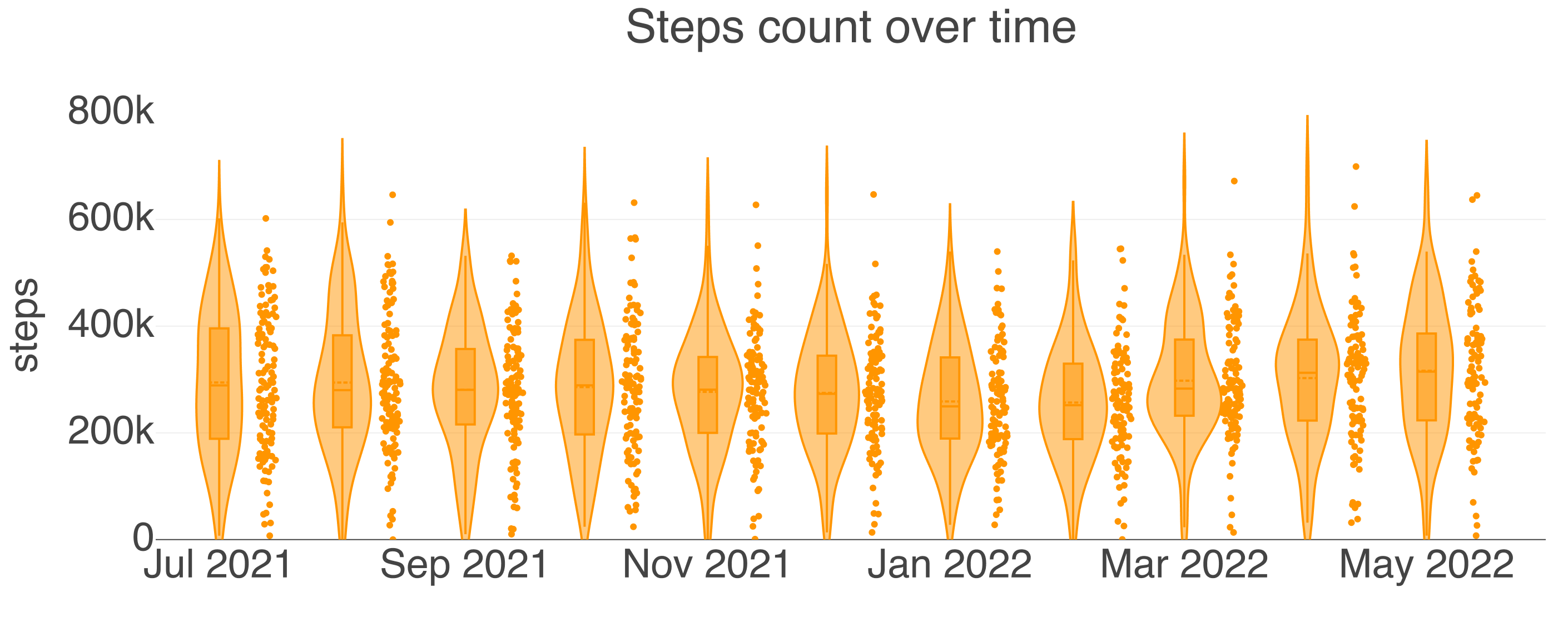

Supplement: ocad140_Supplementary_Data [file ocad140_supplementary_data.zip › ocad140_Supplementary_Data/fig_1_step_count.png]

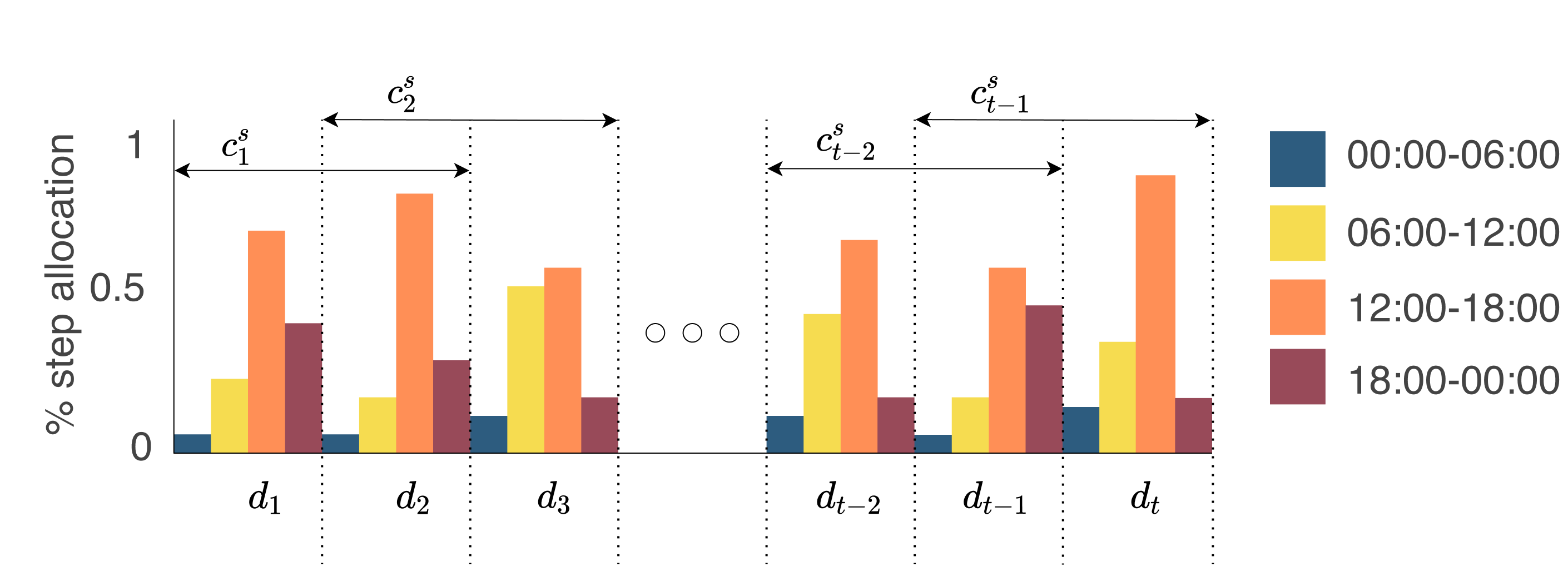

Supplement: ocad140_Supplementary_Data [file ocad140_supplementary_data.zip › ocad140_Supplementary_Data/fig_2_schema_dist_short_term.png]

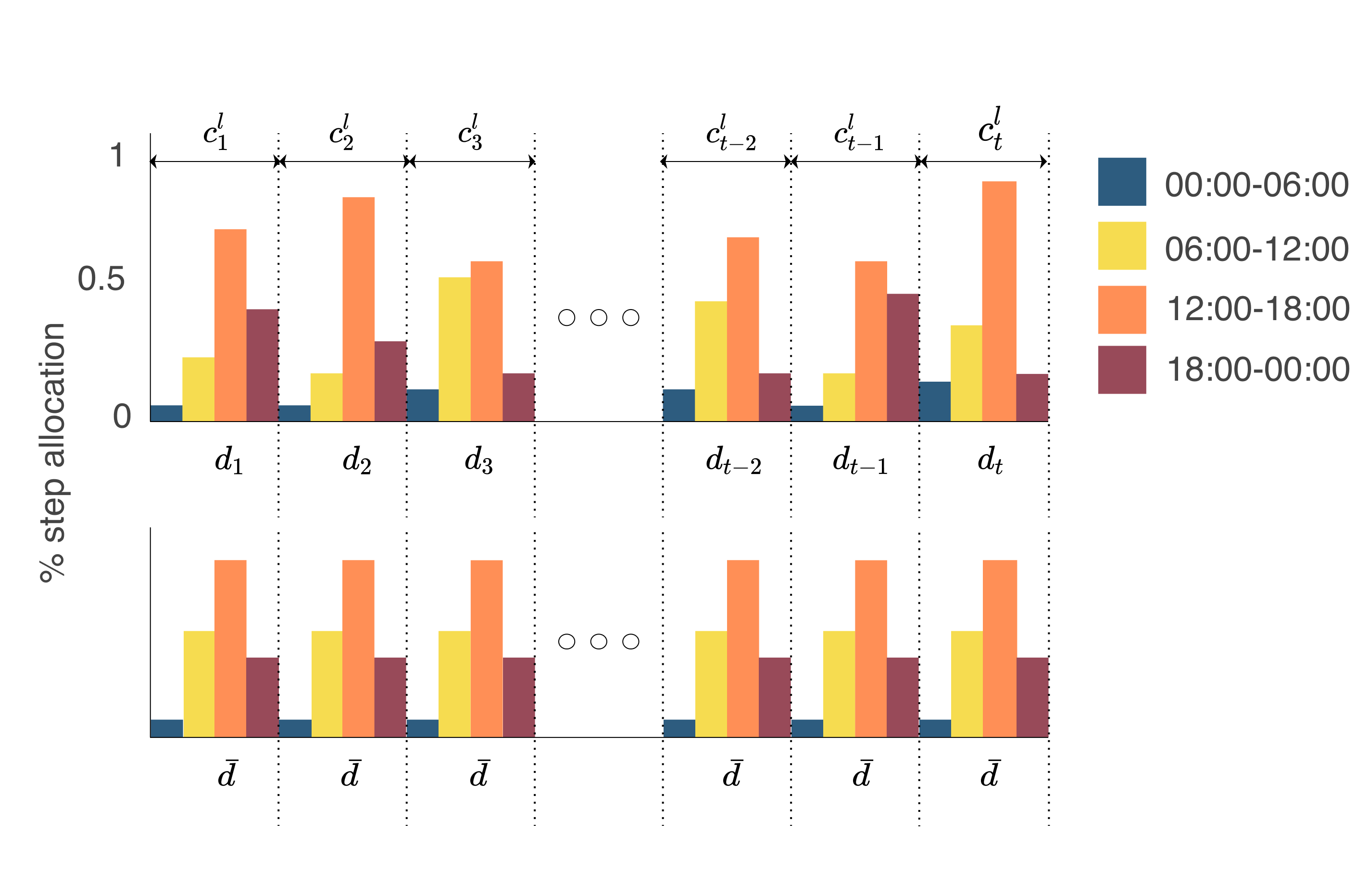

Supplement: ocad140_Supplementary_Data [file ocad140_supplementary_data.zip › ocad140_Supplementary_Data/fig_3_schema_dist_long_term.png]

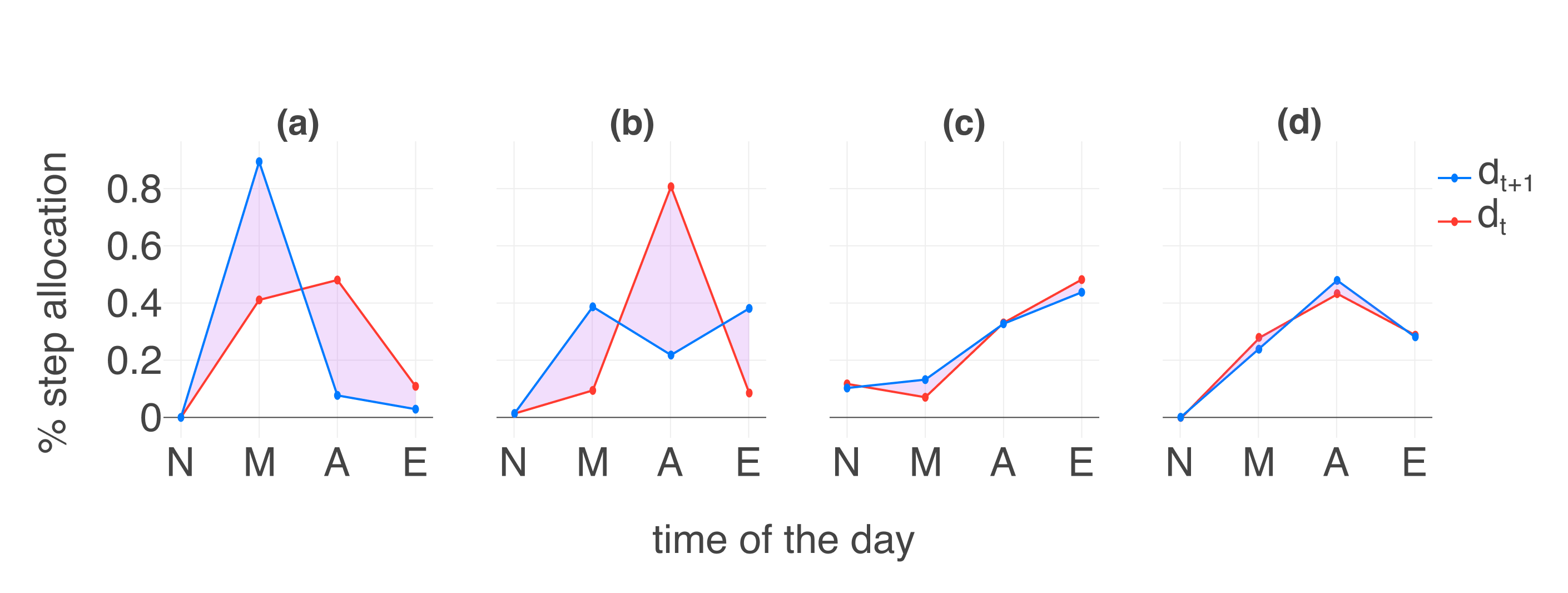

Supplement: ocad140_Supplementary_Data [file ocad140_supplementary_data.zip › ocad140_Supplementary_Data/fig_4_lh_consistency.png]

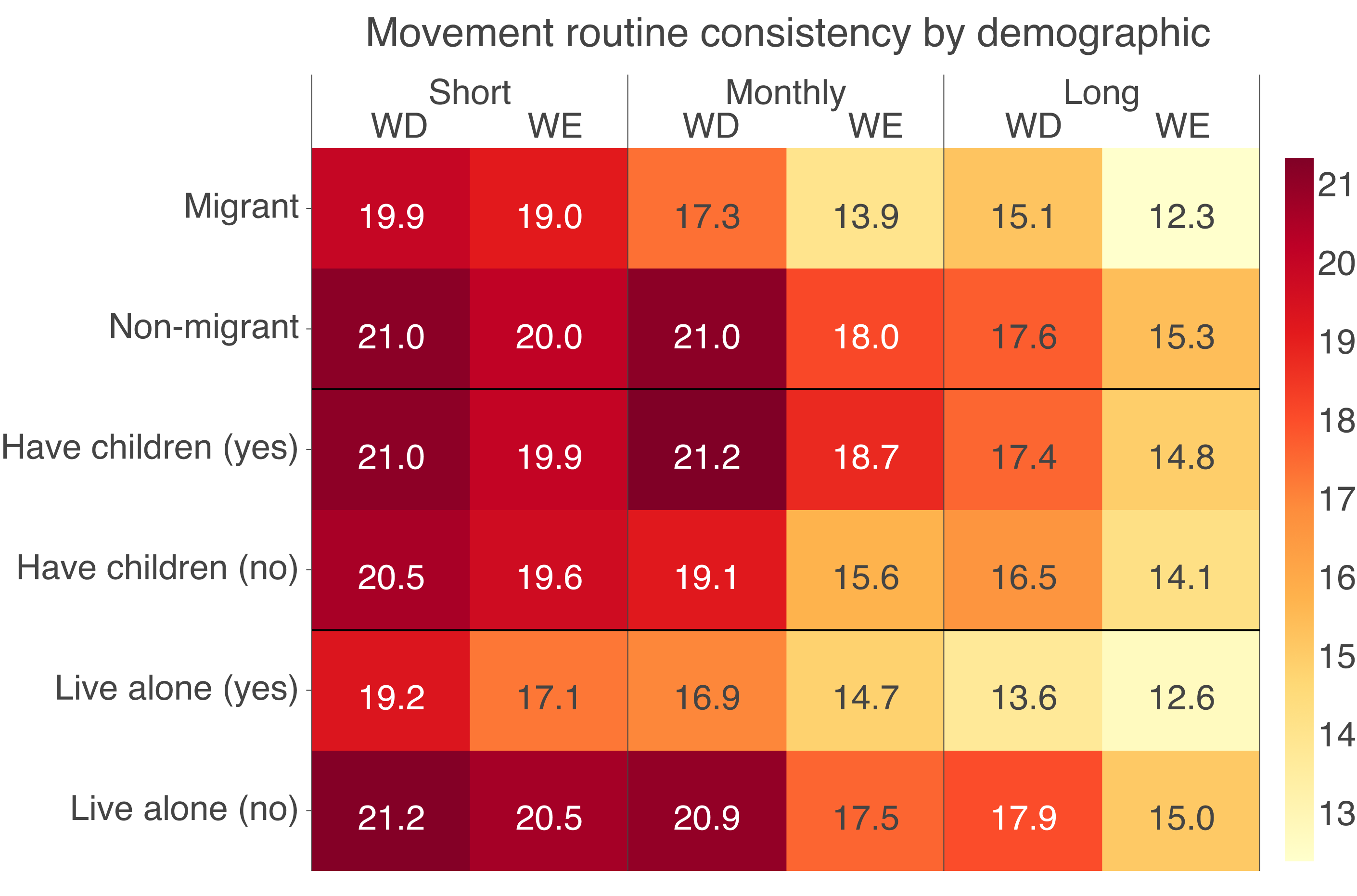

Supplement: ocad140_Supplementary_Data [file ocad140_supplementary_data.zip › ocad140_Supplementary_Data/fig_5_demographic_consistency.png]

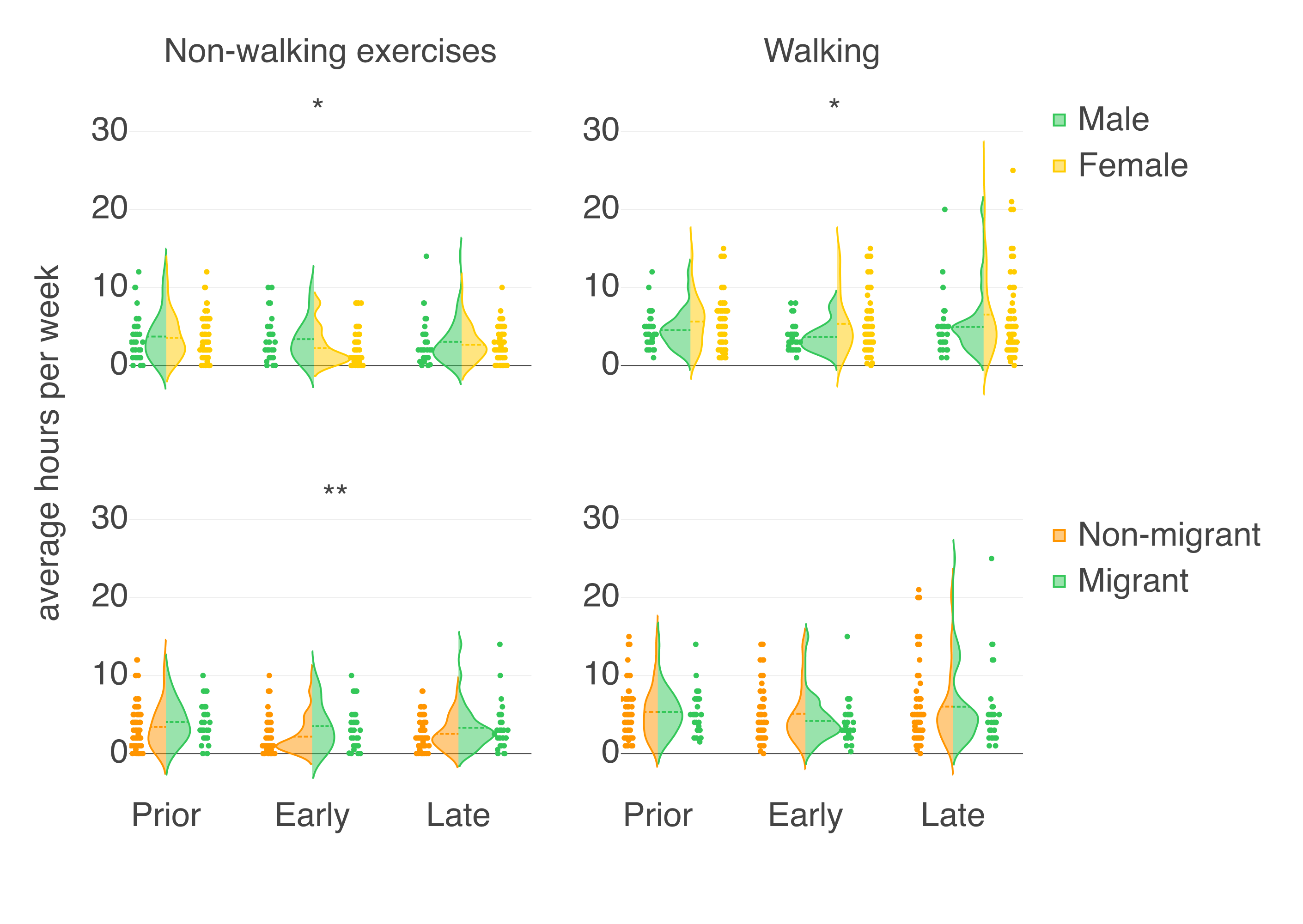

Supplement: ocad140_Supplementary_Data [file ocad140_supplementary_data.zip › ocad140_Supplementary_Data/fig_6_activities_comparison.png]
